# Reducing the environmental impact of trials: a comparison of the carbon footprint of the CRASH-1 and CRASH-2 clinical trials

**DOI:** 10.1186/1745-6215-12-31

**Published:** 2011-02-03

**Authors:** Saleena Subaiya, Euan Hogg, Ian Roberts

**Affiliations:** 1 Public Health and Environment, London School of Hygiene and Tropical Medicine, Keppel Street, London, UK; 2 Edinburgh Centre for Carbon Management, Tower Mains Studios 18F Liberton Brae Edinburgh, UK; 3 CRASH Trials Coordination Centre, London School of Hygiene and Tropical Medicine, Keppel Street, London, UK

## Abstract

**Background:**

All sectors of the economy, including the health research sector, must reduce their carbon emissions. The UK National Institute for Health Research has recently prepared guidelines on how to minimize the carbon footprint of research. We compare the carbon emissions from two international clinical trials in order to identify where emissions reductions can be made.

**Methods:**

We conducted a carbon audit of two clinical trials (the CRASH-1 and CRASH-2 trials), quantifying the carbon dioxide emissions produced over a one-year audit period. Carbon emissions arising from the coordination centre, freight delivery, trial-related travel and commuting were calculated and compared.

**Results:**

The total emissions in carbon dioxide equivalents during the one-year audit period were 181.3 tonnes for CRASH-1 and 108.2 tonnes for CRASH-2. In total, CRASH-1 emitted 924.6 tonnes of carbon dioxide equivalents compared with 508.5 tonnes for CRASH-2. The CRASH-1 trial recruited 10,008 patients over 5.1 years, corresponding to 92 kg of carbon dioxide per randomized patient. The CRASH-2 trial recruited 20,211 patients over 4.7 years, corresponding to 25 kg of carbon dioxide per randomized patient. The largest contributor to emissions in CRASH-1 was freight delivery of trial materials (86.0 tonnes, 48% of total emissions), whereas the largest contributor in CRASH-2 was energy use by the trial coordination centre (54.6 tonnes, 30% of total emissions).

**Conclusions:**

Faster patient recruitment in the CRASH-2 trial largely accounted for its greatly increased carbon efficiency in terms of emissions per randomized patient. Lighter trial materials and web-based data entry also contributed to the overall lower carbon emissions in CRASH-2 as compared to CRASH-1.

**Trial Registration Numbers:**

CRASH-1: ISRCTN74459797

CRASH-2: ISRCTN86750102

## Background

In November 2008, England became the first country in the world to establish a legally binding agreement to reduce carbon emissions by 80% from 1990 levels by 2050 [1]. This requires action to be taken by all sectors of the economy, including the health and health research sectors. The National Institute for Health Research (NIHR) and the Universities that conduct NIHR funded research are therefore required to reduce their carbon footprint. The need for caution in relation to the environmental impacts of medical research is also stated in the Declaration of Helsinki [2]. The NIHR has recently drafted guidelines for researchers to improve the carbon efficiency of their clinical research (http://www.nihr.ac.uk/publications/Pages/carbon_reduction_guidelines.aspx, Table 1).

**Table 1:** NIHR Guidelines for Carbon Reduction in Clinical Trials^1^

Setting the research question and full use of existing evidence	• Always carry out systematic reviews of existing evidence before new grant submission. • Involve clinicians and patients in shaping applied research agendas.
Efficient study design	• Efficient use of resources such as patient populations and patient time. • Consider the possibility of answering several questions through one study. • Involve methodologists in research design.

Study set up	• Maximize professional assistance through research funders, clinical trial units, and NIHR networks to minimize time spent on bureaucratic process. • Decrease time spent on patient recruitment.

Avoid unnecessary data collection	• Clear study protocols. • Measure outcomes remotely when possible (phone, internet). • If patient contact is necessary, utilize outcome assessors that are in close proximity to patients. • Do not measure all outcomes on all patients if unnecessary.

Sensible clinical trial monitoring	• Focus on issues that are critical for safety and wellbeing of study participants and reliability of results. • Avoid monitoring that requires extensive travel to study sites; limit travel to sites only when there is an issue or concern. • Use centralized, systematic programs to ensure data authenticity and quality.

Good practice in research reporting	• Report results of new primary research within updated systematic reviews of other relevant research. • Ensure research reports are presented in a manner that allows information to be utilized by readers.

^1 ^The NIHR Carbon Guidelines, (I. Roberts, personal communication, 20 October 2010)

The CRASH-1 and CRASH-2 trials were international multi centre randomized controlled trials conducted by the London School of Hygiene & Tropical Medicine (LSHTM). The CRASH-1 trial (April 1999- May 2004) evaluated the effect of corticosteroid administration on patient outcomes after traumatic brain injury. The CRASH-2 trial (May 2005- February 2010) examined the effect of tranexamic acid administration in bleeding trauma patients. The two trials were of similar design but greater effort was made to reduce the carbon footprint of the CRASH-2 trial using several of the strategies outlined in the NIHR carbon reduction guidelines. In this study we compared the greenhouse gas emissions of the CRASH-1 and CRASH-2 clinical trials.

## Methods

A carbon audit was conducted of the CRASH-1 and CRASH-2 clinical trials in collaboration with the Edinburgh Centre for Carbon Management (ECCM). The ECCM has completed over 1,100 greenhouse gas quantifications for public and private sector bodies. For each trial, carbon emissions were calculated for a one-year period corresponding to steady state patient recruitment for each trial (CRASH-1: August 2003 to July 2004, CRASH-2: August 2008 to July 2009). Data were collected on all trial elements that would generate carbon emissions according to the greenhouse gas reporting protocol developed by the World Business Council for Sustainable Development (WBCSD) [3] (Table 2). Greenhouse gas emissions were estimated using conversion factors from the Department for Environment, Food and Rural Affairs, Intergovernmental Panel on Climate Change (IPCC), the Swiss Centre for Lifecycle Inventories (SCLCI) and the UK Department for Business, Enterprise and Regulatory Reform (BERR) [4-9]. The global warming potential in carbon dioxide equivalents for methane and nitrous oxides were calculated using conversion factors from the IPCC third annual report [5]. Distances for business travel and transport of study equipment were estimated using a web-based mileage calculator [10] for travel between major cities and World Airport Codes distance calculator [11] for smaller airports within a country. A sample calculation of carbon emissions due to air travel is provided (Table 3).

**Table 2:** Trial activities included in the carbon audit

*Coordination Centre*	• Electricity • Natural Gas • Steam • Heating Oil • Water • Waste
*Trial Related Travel*	• Train • Flights • Accommodation

*Trial Team Commuting*	• Train • Bus • Underground

*Freight Delivery*	• Air freight • Diesel Van

**Table 3:** Sample calculation of trial related travel due to short-haul flights in the CRASH-1 trial

Total short-haul distance travelled (km/yr)	Uplift factor (%)	Emissions for short-haul flights	CO_2 _emitted (t/yr)
30,249 X	109 X	0.098 kgC02/pass.km= 0.001gCH4/pass.km= 0.004 gN20/pass.km=	3.2 + 3.3 × 10^-5 ^+ 1.3 × 10^5^
			**Total = 3.3**

The carbon emissions due to energy consumption (electricity, natural gas, heating oil and steam) and water by the coordinating centre premises could not be calculated directly. This was because of the open plan form of the trial offices and the fact that other non-trial activities were carried out in the same area during the conduct of both trials. Emissions were therefore estimated on the basis of average per person emissions from office space and the number of trial employees (9 in CRASH-1 and 7 in CRASH-2). Electricity consumption of the trial team was based upon percentage of total electricity consumed by the trial office.

Carbon dioxide equivalents are reported separately for CRASH-1 and CRASH-2 by source of emissions, including emissions from coordination centre, trial related travel, commuting and drug box shipments. Emissions per patient were calculated by dividing each trial's total greenhouse gas emissions estimated from the audit year by the total number of patients randomized.

## Results

The total emissions in carbon dioxide equivalents during the one-year audit period were 181.3 tonnes for CRASH-1 and 108.2 tonnes for CRASH-2 (Table 4). Patient recruitment took 5.1 years for the CRASH-1 trial and 4.7 years for the CRASH-2 trial. Assuming the audit year is representative of total trial emissions, CRASH-1 was responsible for 924.6 tonnes of carbon dioxide equivalents and CRASH-2 for 508.5 tonnes. In CRASH-1 this would be equivalent to 681 round trip flights from London to New York for one passenger, and in CRASH-2 this would be equivalent to 374 round trip flights. Overall, CRASH-2 emitted 45% less carbon than CRASH-1.

**Table 4:** Summary of Greenhouse Gas emissions by trial activity for the CRASH-1 and CRASH-2 trials

Source of Emissions	CRASH-1 (tonnes of C0_2 _equivalents/yr)	CRASH-2 (tonnes of C0_2 _equivalents/yr)	Percentage difference in emissions (%)
Electricity	29.0	23.0	-21
Natural Gas	13.0	9.7	-25
Heating Oil	7.6	5.9	-23
Steam	0.2	0.1	-50
Water	0.3	0.2	-33
Waste	4.5	0.6	-87
**Coordination centre total**	**54.6**	**39.5**	**-28**
Train travel	0.7	0.2	-71
Air travel	28.0	29.0	+3
Accommodation	8.6	1.9	-78
**Trial related travel total**	**37.3**	**31.1**	**-17**
Commuting-Train	0.7	1.0	+43
Commuting- Bus	1.3	0.4	-69
Commuting- Underground	1.4	2.0	+43
**Trial team commuting total**	**3.4**	**3.4**	**0.0**
Freight deliveries- Diesel vans	5.0	5.2	+4.0
Freight deliveries- Air freight	81.0	29.0	-64
**Freight delivery total**	**86.0**	**34.2**	**-60**
**Total Trial Emissions**	**181.3**	**108.2**	**40**

**Figure 1:**
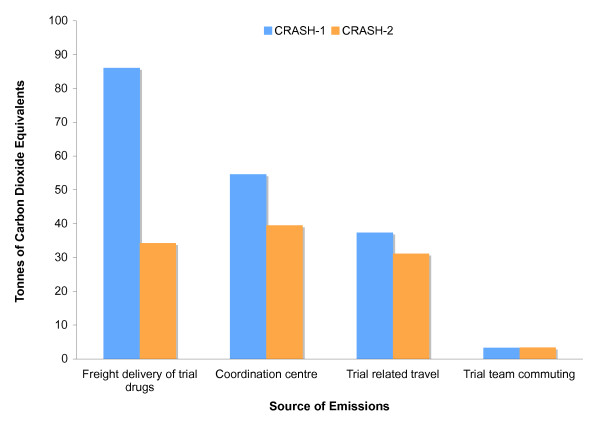
**Comparison of greenhouse gas emissions by activities in the CRASH-1 and CRASH-2 trials**.

The CRASH-1 trial recruited 10,008 patients, corresponding to 92 kg of carbon dioxide per randomized patient. The CRASH-2 trial recruited 20,211 patients, corresponding to 25 kg of carbon dioxide per randomized patient. CRASH-2 emitted 73% less carbon per randomized patient than CRASH-1 (Figure 1).

### Freight delivery of trial drugs

Freight delivery was the largest source of emissions for the CRASH-1 trial (86.0 tonnes, 48% of total emissions). It was the second largest source of emissions for the CRASH-2 trial (34.2 tonnes, 32% of total emissions). Air-transportation of treatment packs comprised the majority of freight emissions in both trials contributing 81.0 tonnes in CRASH-1 (94% of freight delivery emissions) and 29.0 tonnes in CRASH-2 (85% of freight delivery emissions). Emissions due to freight delivery emissions were 60% less in CRASH-2 as compared to CRASH-1.

### Coordination centre

The coordination centre was the second largest source of emissions for the CRASH-1 trial (54.6 tonnes, 30% of total emissions). It was the largest source of emissions for the CRASH-2 trial (39.5 tonnes, 37% of total emissions). Electricity use comprised the greatest proportion of emissions of the coordination centre and contributed 29.0 tonnes during CRASH-1 (53% of coordination centre emissions) and 23.0 tonnes during CRASH-2 (58% of coordination centre emissions). In both trials, the second largest proportion of emissions in this category was due to natural gas consumption. Overall, there was 28% less carbon emitted due to the coordination centre from CRASH-1 to CRASH-2.

### Trial related travel

Trial related travel accounted for 37.3 tonnes (21% of total emissions) in CRASH-1 and 31.1 tonnes (29% of total emissions) in CRASH-2. Twenty-eight tonnes (75% of travel emissions) were due to air-travel in CRASH-1, and 29.0 tonnes (93% of travel emissions) were due to air-travel in CRASH-2. The remainder of carbon emissions in both trials was due to hotel stays. There was 17% less carbon emitted from CRASH-1 to CRASH-2 due to trial related travel.

### Trial team commuting

Commuting accounted for 3.3 tonnes of emissions in both CRASH-1 and CRASH-2. The majority of emissions in both trials were due to underground tube travel, contributing 1.4 tonnes (42% of commuting emissions) in CRASH-1 and 2.0 tonnes (61% of commuting emissions) in CRASH-2.

## Discussion

The results of this comparative carbon audit show that the CRASH-2 trial was considerably more carbon efficient than CRASH-1, both in regards to the amount of carbon emitted per randomized patient and in terms of total emissions. The main reason was that the CRASH-2 trial recruited more than twice as many patients in a shorter time frame than CRASH-1. This in turn was facilitated by the strong clinician interest in the research question, the use of an established network of international collaborators and the use of a simple trial design with data collection limited to key clinically relevant end points. The more efficient recruitment in the CRASH-2 trial helped to minimize carbon emissions per randomized patient.

Differences in the emissions arising from the delivery of trial drugs accounted for a large part of the difference in total carbon emissions between the trials. The trial treatment in CRASH-2 was lighter and more compact than in CRASH-1, weighing approximately 2.5 kg and 9 kg respectively. Each treatment pack in the CRASH-1 trial contained 11 glass vials approximately 5 cm in height and width that had to be protected from breakage during transit by means of protective packaging. On the other hand, the CRASH-2 treatment pack contained four small ampoules that required less protection. Despite a larger number of delivery sites in CRASH-2, emissions were nevertheless reduced in this sector illustrating how logistics can have an important impact on carbon emissions.

Emissions from the coordination centre were slightly lower for CRASH-2 than for CRASH-1. The carbon emissions from the coordinating centre were based on the number of office staff and there were fewer staff in CRASH-2 than in CRASH-1. In the CRASH-1 trial, handwritten data received by fax were double entered by two staff members at the coordination centre. CRASH-2 used direct data entry, which reduced the need for data entry staff as well as reducing the risk of transcription errors.

Trial-related travel often comprises an important source of emissions in clinical trials and limiting unnecessary travel can reduce their carbon footprint [12]. Greater use of statistical data checking algorithms has been suggested as a strategy to reduce the need for on-site data monitoring and these methods were employed in both trials [13]. Additionally, teleconferencing, video-conferencing and web based training materials can be used to reduce travel.

### Strengths and limitations

This study collected information on all sources of carbon emissions for two large multi centre trials. The same methodology was used to determine carbon emissions for CRASH-1 and CRASH-2, helping to ensure a valid comparison between trials. Furthermore, the audit was carried out by an independent, third party organization with extensive experience in quantifying and verifying GHG emissions from operational activities.

However, our study has limitations. The audit period was for only one year and extrapolation to the entire trial may have led to inaccuracy. Data were collected retrospectively, whereas prospective data collection would have been more accurate. The energy consumption of the coordination centre was not obtained by direct measurement, which could lead to errors in estimation of the true carbon emissions. Although the most up-to-date standardized carbon conversion factors were used, the science behind these equivalents is still developing and estimates may not represent actual carbon dioxide equivalent emissions. However, since the same conversion factors were applied to both trials, the comparison between trials and demonstrated reduction is internally valid.

## Conclusions

This study has shown that the efficiency of patient recruitment is a major determinant of the environmental impact of a clinical trial. There is a limit to the amount of greenhouse gases that can be emitted without causing serious climate destabilization. If society decides to allocate a proportion of its limited carbon budget to obtaining information on the safety and effectiveness of a health care intervention, then both researchers and research regulators have the responsibilities to ensure that the research is conducted as efficiently as is consistent with patient safety and that it provides valid and reliable information.

In the UK, there is a growing consensus that the regulation and governance framework for clinical trials has become an obstacle to their efficient conduct [14]. The Academy of Medical Sciences has been commissioned by the UK Government to undertake an independent review of the regulation and governance of medical research [15]. Although the CRASH-2 trial recruited patients more rapidly than its predecessor, this was largely driven by recruitment in Asia, South America and Africa, while recruitment in the UK was much less than anticipated largely due to these regulatory changes implemented after CRASH-1 [16]. Streamlining and improving the regulatory environment has the potential to increase both the carbon and cost effectiveness of medical research.

Although efforts to reduce the carbon footprint of clinical trials are essential, it is important to acknowledge that healthcare itself is a major source of carbon emissions and that by ensuring the provision of safe and effective healthcare, health research plays a central role in avoiding medical waste. Pharmaceutical use is particularly carbon intensive and makes a major contribution to the carbon footprint of the NHS. The CRASH-1 trial showed that a drug that had been used in the treatment of head injury patients for over thirty years was at best ineffective, and probably harmful [17]. The carbon cost of obtaining reliable information from well-conducted clinical studies is likely to be dwarfed by that of using treatments that are ineffective or harmful.

In summary, a number of strategies can be used to reduce the carbon footprint of clinical research. Although it is not always possible to reduce the weight and shipping distance of trial materials, the use of lightweight packaging can have an important impact on emissions. Statistical data monitoring, web-based training materials and tele-conferencing can help reduce travel. Direct data entry can reduce the need for transcription and will also lessen emissions from trial office space. While the focus of this study was on emissions from non-commercial clinical trials, the extent to which the principles might be applied in pharmaceutical industry directed trials is open to question. Industry led trials are often considerably more complex and involve much more data collection than non-commercial trials. On the other hand, it is likely that the carbon footprint of industry led trials is much larger, and the need to make emissions reductions even more important.

## Competing interests

The authors declare that they have no competing interests.

## Authors' contributions

SS participated in coordination of the study and drafted the manuscript

EH conducted the carbon audit of trial related activities.

IR conceived of the study and participated in its design and coordination and helped to draft the manuscript.

All authors read and approved the final manuscript.
